# Aerobic Exercise Versus Plyometrics in Reducing Anxiety Levels in College Students With Mild Generalized Anxiety Disorder

**DOI:** 10.7759/cureus.70165

**Published:** 2024-09-25

**Authors:** Aishwarya S, Priyadharshini Kumar

**Affiliations:** 1 Physiotherapy, Saveetha College of Physiotherapy, Saveetha Institute of Medical and Technical Sciences, Saveetha University, Chennai, IND

**Keywords:** aerobic exercise, beck anxiety inventory, general anxiety disorder, physical activity, plyometrics

## Abstract

Background

Generalized anxiety disorder is one of the most prevalent mental disorders, characterized by excessive anxiety and worry that affect both mental and physical health. These pervasive illnesses have a crippling effect on people's everyday lives, quality of life, and wellness and are also highly linked with cardiovascular risk factors. Physical activity-based therapies have shown promising effects in treating a range of mental disorders, from psychosis to dementia. Out of many conventional therapies, aerobic exercise and plyometrics have been found to be effective in reducing anxiety levels.

Aim

This study aims to compare the effectiveness of aerobic exercise and plyometrics in reducing anxiety levels in college students with mild generalized anxiety disorder.

Materials and methods

A total of 96 subjects were selected for the study using a stratified sampling technique; 48 subjects were assigned to the aerobic exercise group (Group A) and 48 to the plyometric group (Group P). Randomization was done using the sealed envelope method. This study includes students aged 18-25 years, both genders, with a body mass index of <30, a Beck Anxiety Inventory (BAI) score of 8-15, and at least six months without practicing any kind of physical activity. The exclusion criteria are subjects with other psychotic disorders, a history of cardiovascular disease, chronic kidney disease, inflammatory disease, malignant conditions, neurological disorders, anemia, with comorbid conditions, who were involved in supportive therapy for anxiety such as cognitive behavioral therapy, use of anxiolytic drugs, females with menstrual disorders, and subjects with recent trauma or injuries (fracture, ligament sprain, or muscle strain). The treatment duration for both groups was three days per week for a total of four weeks. Group A consists of five minutes of warm-up, 30 minutes of walking, and five minutes of cool-down, for a total of 40 minutes. Group P consists of warm-up of five minutes, high knees, single leg hop and jump squats of two sets and 10 repetitions for 30 minutes, and a cool-down of five minutes, for a total of 40 minutes.

Conclusion

The anxiety levels were analyzed using the BAI, revealing that both groups have shown improvements in Beck anxiety scores; however, Group A has shown a comparatively more significant improvement than Group P.

## Introduction

Anxiety disorders are a diverse group of prevalent mental health conditions typified by hyperarousal, intense fear, and worry. While anxiety is a common feature of anxiety disorders, the symptoms might vary depending on the particular subtype of the condition. Subcategories of anxiety disorders comprise generalized anxiety disorder (GAD), social phobia, panic disorder, specific phobias, agoraphobia, separation anxiety disorder, and selective mutism. Although anxiety and depression are equally common, the former has received less attention and is frequently misdiagnosed and treated poorly [1].

One of the most widespread mental illnesses affecting young people is anxiety. Based on statistics from 2017, 284 million people globally, or 3.8% of the global population, struggle with anxiety disorders. The prevalence of anxiety disorders is estimated to differ by nation globally, with estimates of up to 70% in those with long-term medical issues [2]. Additionally, anxiety disorders rank as the sixth most prevalent cause of disability worldwide, per statistics from the Global Burden of Disease survey [3]. To lessen their stress, people with anxiety disorders sometimes adhere to undesirable coping techniques such as substance misuse or avoidance [4]. Tragically, there is evidence connecting these conditions to higher cardiovascular risk factors such as high blood pressure and early mortality [5]. These widespread illnesses severely impair people's well-being, quality of life, and day-to-day activities [6].

Among mental disorders, GAD is one of the most prevalent ones. Often, excessive worry or anxiety persists most of the days or at least six months over a variety of topics, including jobs, social interactions, personal health, and day-to-day routines. Symptoms include feeling tense, easily exhausted, having trouble focusing, having a blank mind, being irritable, having trouble managing worry, restlessness, and having unpleasant sleep. It also has an effect on physical symptoms such as loose stools, excessive perspiration, tense muscles, nausea, and vomiting. The leading causes stem from a complex interaction between biological and environmental factors, which can include heredity, developmental disparities, threat perception abnormalities, and changes in brain chemistry and function. GAD is frequently associated with suicidal thoughts and self-medication with alcohol or other medications [7,8]. Unfortunately, there has not been much discussion about mental health issues such as anxiety, which adds to the large proportion of people who suffer from anxiety but are left undiagnosed [9].

Pharmacotherapy and cognitive behavioral therapy (CBT), or a combination of these, are frequently used to treat anxiety disorders, although up to one-third of patients may not benefit from these conventional methods, which results in treatment dropout, poor results, and impaired functioning [10,11]. Physical activity (PA)-based therapies symbolize a unique strategy that has shown potential in addressing symptoms of a variety of mental health disorders, from psychosis to dementia [12]. It has also been repeatedly proven to have an antidepressant effect on depressed individuals [13]. According to recent research, PA may also be helpful in the treatment of anxiety disorders as well and can effectively reduce symptoms in people both diagnosed and undiagnosed with anxiety disorders as a stand-alone or adjunctive therapy [14-16]. Lowering cardiovascular risks is one of PA's most important features. Conversely, one known risk factor for the emergence of anxiety is physical inactivity [17,18].

It has been discovered that aerobic exercise is a helpful and efficient therapeutic substitute for a variety of anxiety and mood disorders [19]. Walking is one of the aerobic activities that has been shown to improve quality of life, mental problems, and aerobic physical fitness [20]. On the other hand, plyometric training is a type of exercise that increases power and speed by imitating the pace and movement patterns of the targeted sport. The term "plyometric" is currently used to describe exercises that originated in "Europe," where they were originally referred to as "jumping training." Evidence also supports that plyometric training has dramatically decreased psychological factors such as stress and anxiety [21]. This study aims to compare the effectiveness of aerobic exercise and plyometrics on reducing anxiety levels and further address shortcomings of previous research by measuring both physical and mental health outcomes [22].

## Materials and methods

The study was ethically approved by the Institutional Scientific Review Board with an ISRB number: 01/001/2023/ISRB/SR/SCPT. This experimental study was conducted at Saveetha Institute of Medical and Technical Sciences in Chennai, India, from October 2023 to December 2023. A total of 96 college students were recruited using the Beck Anxiety Inventory (BAI) questionnaire with mild GAD meeting the inclusion criteria and were clarified about the safety of the treatment and the ease of the process. Additionally, written consent was obtained from the participants. By stratified random sampling, the willing participants were assigned into two groups, Group A (the aerobic exercise group, n = 48) and Group P (the plyometric exercise group, n = 48), and randomized by the sealed envelope method. The outcome assessors were blinded to group allocation to ensure unbiased data collection. The intervention period for both groups was three days per week for four weeks. Both interventions were home-based exercise programs with a weekly follow-up of the participants. This study includes students aged 18-25 years, both genders, with a body mass index of <30, a BAI score of 8-15, and at least six months without practicing any kind of PA. The exclusion criteria are subjects with other psychotic disorders, a history of cardiovascular disease, chronic kidney disease, inflammatory disease, malignant conditions, neurological disorders, anemia, with comorbid conditions, who were involved in supportive therapy for anxiety such as CBT, use of anxiolytic drugs, females with menstrual disorders, and subjects with recent trauma or injuries (fracture, ligament sprain, or muscle strain).

Outcome measure

BAI Scale

This scale contains 21 self-reported items, with a Likert scale ranging from 0 to 3, with a total score range of 0 to 63, used to measure common somatic and cognitive symptoms of anxiety. It was found to have high internal consistency and test-retest reliability and good concurrent and discriminant validity. BAI was able to discriminate homogeneous and heterogeneous anxious diagnostic groups from other psychiatric groups. BAI scores are classified as minimal (0-7), mild (8-15), moderate (16-25), and severe (26-63) [23].

Procedure

Group A (Aerobic Exercise Group, n=48)

This program consists of performing four weeks of low-intensity aerobic exercise in a park with a flat terrain or terrace, three days per week, with a total duration of 40 minutes. The participant was asked to select a suitable ambiance and fitting shoes for walking and had to perform a warm-up of five minutes, a walking period of 30 minutes, and a cool-down period of five minutes. Warm-up and cool-down consist of general body stretching. They are advised to take a rest in between if they want to and continue the session.

Group P (Plyometric Exercise Group, n=48)

This program consists of performing four weeks of low-intensity plyometric exercise, three days per week in a suitable ambiance. The warm-up phase is five minutes, the training phase is 30 minutes with a rest period of three minutes between the sessions, and the cool-down phase is five minutes, for a total duration of 40 minutes. The training phase consists of three exercises: high knees, single leg hops, and jump squats of all two sets and 10 repetitions.

High knees: Stand straight with your feet at hip width and arms at the sides, with the weight of the body evenly distributed to both legs. Raise your left arm and bend your elbow while simultaneously raising your right knee as high as you can but not lower than the hip level. Quickly swap between your arms and legs like sprinting in place until completing your repetitions.

Single leg hop: Start by standing on one leg and bend the knee of the other leg. Place your hands on the hips to check they are level. Now, start to hop on the standing leg, focussing on a controlled landing. After completing the reps on the standing leg, swift to the other leg and start to hop. Do this for 10 repetitions on each leg for two sets.

Jump squats: Stand with hands at the sides and feet one inch wider than shoulder-width apart. Slightly tilt your hip back and lower into a squat with thighs parallel to the floor by bending your knees. Make sure that your knee does not move forward than feet level while you squat. Look straight and clasp your hands if it is comfortable for you while you squat down. Now, leap off the ground from the lowest point of your squat. You can put your arms by your sides while jumping. Continue at a normal pace so that you have a smooth landing.

Statistical analysis

Analysis was done by IBM SPSS Statistics for Windows, Version 28 (Released 2021; IBM Corp., Armonk, New York). The pre-test scores of anxiety levels were measured using BAI and were assessed before the initiation of intervention and measured after four weeks of intervention and taken as post-test values. The collected data were evaluated and tabulated, and the significant differences between pre-test and post-test measures were analyzed using descriptive analysis to find the mean and standard deviation and interferential analysis using unpaired t-test for between-group comparisons and paired t-test for within-group comparisons.

## Results

**Figure 1:**
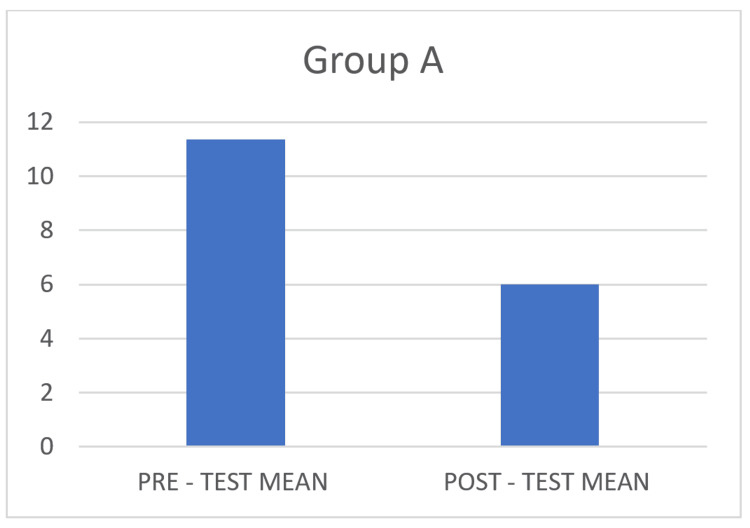
Pre- and post-test mean for Group A

A total of 96 college students were recruited with mild GAD screened using BAI and were equally divided into two groups (48 + 48) meeting the inclusion criteria. Group A was prescribed aerobic exercise and Group P with plyometric exercise, both for a duration of 40 minutes. The pre- and post-test values of BAI for Group A findings showed a significant improvement in reducing anxiety and are considered statistically significant, with a p-value of <0.0001, as shown in Table 1, and a mean difference of 5.35, as depicted in Figure 1.

**Table 1:** Pre- and post-test values of BAI for Group A BAI: Beck Anxiety Inventory

Outcome Measure	Group A	Mean	SD	t-value	p-value
Beck Anxiety Inventory	Pre-test	11.35	2.58	9.7885	<0.0001
	Post-test	6.00	2.78		

**Figure 2:**
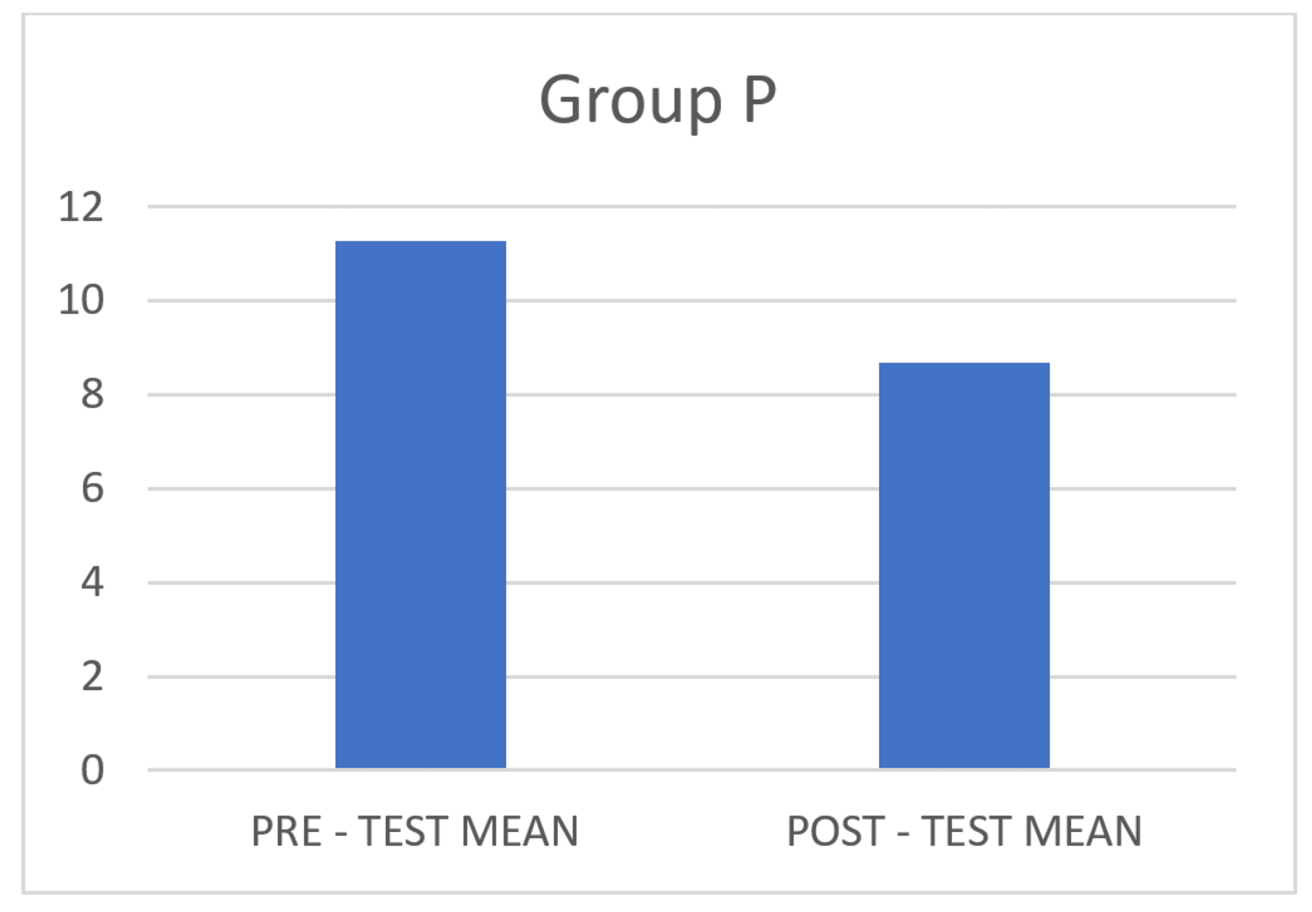
Pre- and post-test mean for Group P

The pre- and post-test values of BAI for Group P findings also showed improvements and are considered statistically significant, with a p-value of <0.0001, as shown in Table 2, and a mean difference of 2.58, as depicted in Figure 2.

**Table 2:** Pre- and post-test values of BAI for Group P BAI: Beck Anxiety Inventory

Outcome Measure	Group P	Mean	SD	t-value	p*-*value
Beck Anxiety Inventory	Pre-test	11.27	2.62	8.8170	<0.0001
	Post-test	8.69	2.60		

**Figure 3:**
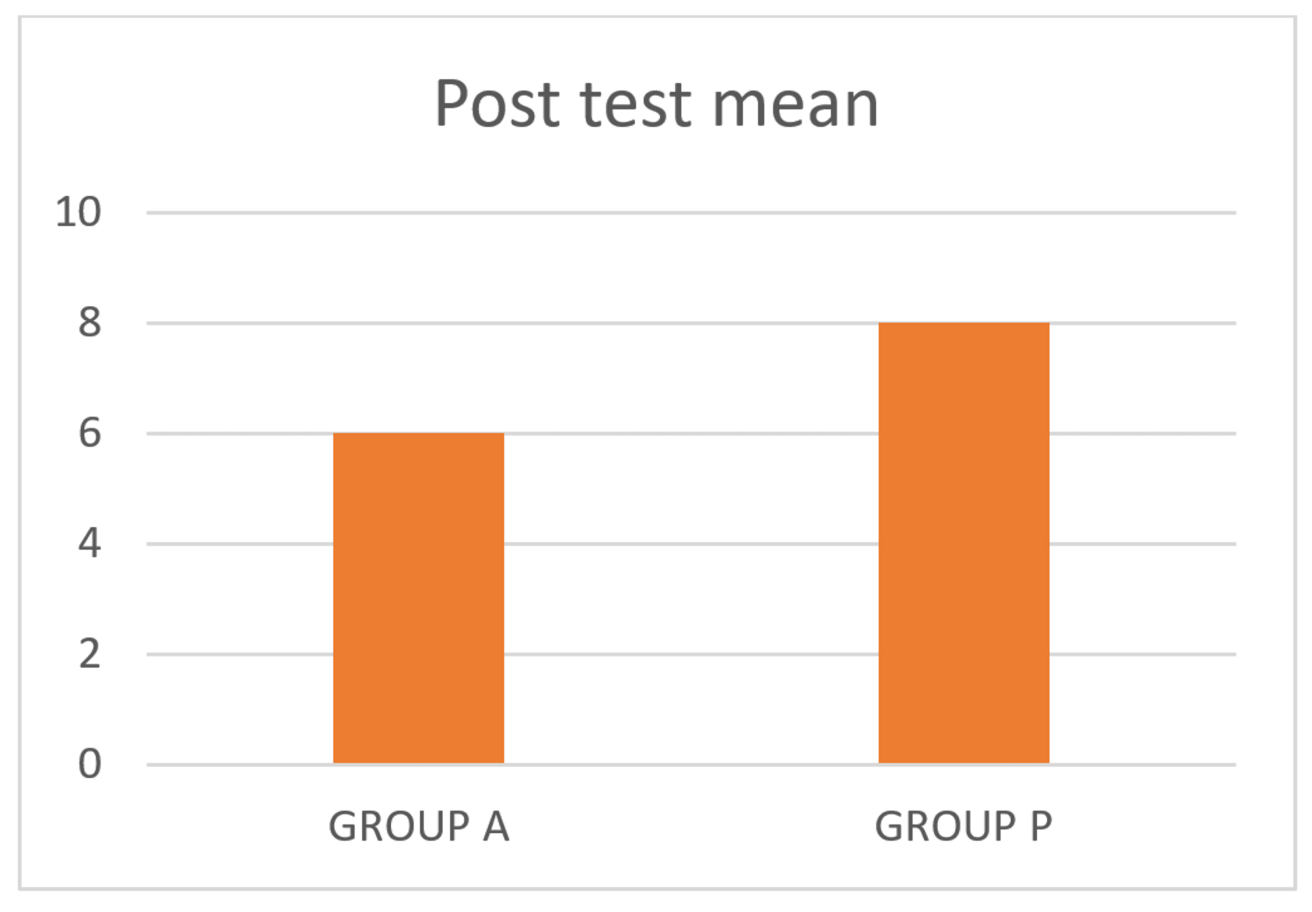
Post-test mean for Group A and Group P

The post-test values of BAI for Group A and Group P findings are considered statistically significant, with a p-value of <0.0001, as shown in Table 3, and a mean difference of 2.69, as depicted in Figure 3. The results suggest that both groups have shown improvement in BAI score with a p-value of <0.0001, but Group A has shown more significant improvement in BAI score than Group P. Therefore, aerobic exercise showed better results when compared to plyometrics in reducing anxiety levels.

**Table 3:** Post-test values of BAI for Group A and Group P BAI: Beck Anxiety Inventory

Outcome Measure	Beck Anxiety Inventory	Mean	SD	t-value	p*-*value
Group A	Post-test	6.00	2.78	4.8940	<0.0001
Group P	Post-test	8.69	2.60		

## Discussion

The purpose of the study was to determine the effectiveness of aerobic exercise and plyometrics on anxiety levels in college students with mild GAD. The duration of the intervention for both groups was four weeks, and the results were measured using BAI. The post-test values of BAI for Group A and Group P were 6.00 and 8.69, respectively, which shows statistically significant differences between groups. Based on statistical analysis, both groups showed improvements in BAI scores, but Group A scores showed significant improvements compared to Group P scores.

A recent study by Philippot et al. in 2022 took a clinical population of 52 adolescent inpatients hospitalized for depression and anxiety in a psychiatry hospital. They were randomly assigned into a physical exercise group and a control group for a duration of three to four times per week over a six-week period. Their study concludes that exercise intervention resulted in significantly reduced symptoms of depression compared to the control social relaxation program and a similar effect on anxiety by both the interventions measured by the Hospital Anxiety Depression Scale. Similarly, this study focuses on providing exercise-based intervention for both groups [24].

A randomized controlled trial by LeBouthillier et al. in 2017 compared a single bout of aerobic exercise versus resistance training by recruiting persons with a diagnosis of anxiety-related disorder. When compared to aerobic exercise, resistance training was effective for a higher number of constructs. Aerobic exercise reduced anxiety, stress, and general psychological distress, while resistance training reduced anxiety sensitivity, symptoms unique to the disorder, distress tolerance, general psychological distress, and intolerance of uncertainty. In contrast, this study compares aerobic exercise versus plyometrics by recruiting students with mild GAD assessed by BAI. But here, although both groups showed improvements in anxiety levels, aerobic exercise showed significant improvement in plyometrics [25].

Similarly, Lattari et al. (2018) conducted a study to check the short-term effects of aerobic exercise on symptoms of anxiety and to investigate the long-term effects of aerobic exercise, aside from alterations in EEG frontal asymmetry, on the intensity and symptoms associated with panic disorder. Anxiety was sharply elevated after exercise and dramatically decreased following a 10-minute recovery period. Regular aerobic exercise was associated with higher reductions in symptoms of panic disorder, BAI, and Beck depression inventory-II. This study showed greater reductions in BAI scores in the aerobic exercise group [26].

Karoni et al. conducted a study in 2021 that compares plyometrics and aerobic exercise on medical students' moods, whereas this study explored anxiety. Six weeks of plyometrics and aerobic exercise exhibited effectiveness in improving mood levels. However, there was not any significant difference between the groups. Thus, they conclude that both interventions have a similar effect in increasing mood scores. On the other hand, the current study proves aerobic exercise to be comparatively effective in improving anxiety levels [27].

Future studies should seek to investigate the effects of both interventions in other population groups, particularly for people with chronic comorbid conditions and hospitalized patients, as they are more prone to get anxiety disorders. Additionally, it should explore other types of anxiety disorders. Exploring additional types of PA or combining exercise with other therapeutic modalities, such as CBT, may have a more comprehensive treatment approach. Future studies could include therapist-supervised intervention protocols to ensure the accuracy and consistency of the results.

Anxiety disorder has a wide array of causes and symptoms so that many other factors may have affected the accuracy of outcome measures before and after the intervention protocol. The researcher should make sure that the symptoms are not temporary and persists for more months. The intervention lasted only four weeks. Anxiety reduction, particularly in clinical populations, may require more extended periods of PA for sustained improvements, so the short duration may not fully reflect the long-term benefits of the interventions. The study included only college students aged 18-25, limiting the applicability of the findings to other age groups or individuals with different life circumstances, such as working adults or older adults.

## Conclusions

This study highlights the efficacy of PA-based therapies in managing anxiety levels among college students with mild GAD. By comparing aerobic exercise and plyometric training, we found that both interventions led to significant reductions in anxiety, as measured by the BAI. However, aerobic exercise was notably more effective in alleviating anxiety symptoms compared to plyometric training. This finding aligns with existing research suggesting that aerobic exercises, such as walking, have a robust impact on mental health by improving cardiovascular function, mood regulation, and overall well-being.

The results underscore the potential of incorporating structured aerobic exercise into anxiety management strategies, particularly for young adults who may benefit from accessible, non-pharmacological treatments. By continuing to investigate and validate exercise-based therapies, we can offer more comprehensive and effective treatment options for individuals struggling with anxiety disorders.
